# Exploring the spatial heterogeneity and temporal homogeneity of ambient PM_10_ in nine core cities of China

**DOI:** 10.1038/s41598-021-88596-8

**Published:** 2021-04-26

**Authors:** Rui Feng, Rong Zhou, Weiwei Shi, Nanjing Shi, Xuekun Fang

**Affiliations:** 1 College of Environmental and Resource Sciences, Zhejiang University, Hangzhou, 310058 People’s Republic of China; 2 State Key Laboratory of Clean Energy Utilization, Zhejiang University, Hangzhou, 310027 People’s Republic of China; 3 Ecological and Environmental Science and Design Institute of Zhejiang Province, Hangzhou, 310012 People’s Republic of China

**Keywords:** Environmental sciences, Environmental chemistry, Environmental impact

## Abstract

We focus on the causes of fluctuations in wintertime PM_10_ in nine regional core cities of China using two machine learning models, Random Forest (RF) and Recurrent Neural Network (RNN). RF and RNN both show high performance in predicting hourly PM_10_ using only gaseous air pollutants (SO_2_, NO_2_ and CO) as inputs, showing the predominance of the secondary inorganic aerosol and implying the existence of thermodynamic equilibrium between gaseous air pollutants and PM_10_. Also, we find the following results. The correlation of gaseous air pollutants and PM_10_ were more relevant than that of meteorological conditions and PM_10_. CO was the predominant factor for PM_10_ in the Beijing-Tianjin-Hebei Plain and the Yangtze River Delta while SO_2_ and NO_2_ were also important features for PM_10_ in the Pearl River Delta and Sichuan Basin. The spatial heterogeneity and temporal homogeneity of PM_10_ in China are revealed. The long-range transported PM_10_ was substantiated to be insignificant, except in the sandstorms. The severity of PM_10_ was attributable to the lopsided shift of thermodynamic equilibrium and the phenology of indigenous flora.

## Introduction

China, the world’s second largest economy, has gone through severe atmospheric deterioration for decades, which have slowed down the economic growth rate and implacably elicited 1.6 million premature deaths in 2017^1^. Air pollution, having elevated daily hospital admissions in 218 cities of China^2^, leads to the increase of cardiopulmonary/cardiovascular diseases, respiratory infection and hypermethylation^3^. Atmospheric particulate matter (PM) draws the most public concerns among air pollutants, because of its toxicity and carcinogenicity^4^. PM can be a host for bacterial and fungal pathogens^5,6^. It has been found that a 1% rise of PM_2.5_ enhances 2.9% of healthcare expenditure in China^7^. Moreover, atmospheric aerosols are possible contributors to weather and climate change^8–11^.

The atmospheric particulate matter with an aerodynamic equivalent diameter of less than 10 μm (PM_10_), whose main emission sources are the coal-based thermal power plants, coal-based domestic heating, automobiles, fugitive dust from roads, construction sites, and unpaved soil^12^, is studied in this work. The reason why we select PM_10_ for investigation is it can greatly affect human’s health, bringing in numerous disease burdens^13–15^. Also, the source of PM_10_ partly originates from long-range transported sandstorms^16^. Investigation on the significance of long-range transport and indigenous emission is of great importance. Several previous works investigated PM in the megacities of China via outdoor observation^17–19^. Machine learning, orchestrated for developing algorithms automatically from large datasets, removes the need for an air pollution emission inventory which is a linchpin for conventional atmospheric models, thus becoming a more flexible approach^20–23^. Compared to inventory-predicated air quality models, machine learning offers an alternative and more accurate method to interpret air pollutant concentration, which now is a popular topic in atmospheric research field. Feng et al.^23^ proposed an avenue to forecast the air pollutants in Hangzhou using machine learning. Chen et al.^24^ used deep neural network to estimate PM_2.5_ concentrations across China. Yan et al.^25^ developed a deep learning model to improve the interpretability and predictive accuracy of satellite-based PM_2.5_. Han et al.^26^ estimated air qualities in Beijing during 2008–2012 by Bayesian Multi-task Long Short-Term Memory. In this work, we select Recurrent Neural Network (RNN) and Random Forest (RF) to conduct a nationwide survey of PM_10_. The concentration of PM_10_ is much higher in winter than in the other seasons, so we focus on the wintertime (December, January and February) PM_10_ in the past more than five years (December 2014 to February 2019).

The scopes of this work are as follows: (1) finding the different regional PM_10_ patterns and its determinants; (2) exploring the contributors of severe wintertime haze in a novel perspective and demonstrating of the insignificance of long-range transport. In Section two, we introduce the study areas, the sources of data, and parameters of two machine learning models. In Section three, we illustrate the causes for severity of haze in wintertime and show the reason why the long-range transported PM_10_ are insignificant except in sandstorms.

## Methods

### Investigated areas

The Beijing-Tianjin-Hebei plain (BTH) (37°–41° N, 114°–118° E), the Yangtze River Delta (YRD) (30°–33° N, 118°–122° E), the Pearl River Delta (PRD) (21.5°–24° N, 112°–115.5° E) and the Si-chuan Basin (SCB) (28.5°–31.5° N, 103.5°–107° E) are the four most prosperous but polluted regions in China, representing the center of North, East, South and West China, respectively. BTH, where the capital city of Beijing and the central municipality of Tianjin nestle, had a population of 110 million and produced over 10% of China’s national gross domestic product (GDP) in 2017. YRD, where the megacity of Shanghai resides, denotes the economic center of China, accounted for 19% of China’s GDP and had a population of 150 million. The PRD urban agglomerations surrounding Hong Kong and Macao created nearly 13% of China’s GDP with a population of 83 million. SCB, the economic and political center of West China, contributed a population of 114 million and 7% national GDP. These four regions comprised 33% of the Chinese population, 8% of China’s land, and 49% of GDP of China in 2017. However, all of these regions have suffered from severe PM_10_ for decades due to the rapid industrialization. In order to develop better control measures, the question emerges as whether the regional patterns of PM_10_ are the same. Because of the regional heterogeneity of natural and anthropogenic sources of PM_10_, a reasonable assumption is the determinants of PM_10_ varies among regions but remains consistent in the same region. Nine regionally representative core cities, which are Beijing and Tianjin in BTH, Shanghai, Nanjing and Hangzhou in YRD, Guangzhou and Shenzhen in PRD, and Chengdu and Chongqing in SCB, are picked to investigate the regional PM_10_ patterns in wintertime. These nine cities, each of which has more than nine million citizens, are the most flourishing areas of China with their ever-growing urbanization. According to census, the permanent residents living in these nine cities were 154 million in 2017.

### Data of wintertime air pollutants and meteorology

All the data used in this work are publicly accessible online. The time period studied was sifted to be wintertime (December, January and February) from 1 December 2014 to 28 February 2019. Hourly air pollutants, including sulfur dioxide (SO_2_), nitrogen dioxide (NO_2_), tropospheric ozone in the surface air (O_3_), carbon monoxide (CO), PM_2.5_ and PM_10_ were extracted from official website of China National Environmental Monitoring Centre (http://beijingair.sinaapp.com/), where the air pollution data from 1563 environmental monitoring sites across China were recorded and documented. We chose the environmental monitoring sites in the nine investigated cities for training and testing. We use the data from all of the environmental monitoring sites in a city to calculate Feature Importance. Then we take the average of them to predict hourly PM_10_ in Scenario one and two. The meteorological data were from the NASA Global Modeling and Assimilation Office (https://gmao.gsfc.nasa.gov/reanalysis/MERRA-2) and the University of Wyoming (http://www.weather.uwyo.edu/surface/meteorogram/seasia.shtml), including hourly temperature, relative humidity, atmospheric pressure, wind speed and wind direction.

### Parameters of random forest and RNN

Recurrent Neural Network (RNN) is capable of capturing temporal contextual information, suitable for simulating the accumulation and deposition of air pollutants. RNN can transfer information from one step to the following step. Random Forest (RF), a tree structuring model, is able to quantitatively rate the significance of each input in shaping the output via calculating the Feature Importance (FI). There are two types of Feature Importance, which are Variable Importance and Gini Importance. In this case, we chose Gini Importance.

Several setups of RF and RNN were tested and fine-tuned before we selected the best settings of parameters. As for RF, n-estimator is the number of built trees. A higher n-estimator ensures the predictions to be stronger and more stable, but also makes the operator code slower. Increasing max-features generally improves the performance of Random Forest, but decreases the diversity of individual tree and slows down the running speed. To strike the right balance, assigning maximum features to be auto to take all features into consideration and put no restriction on the individual tree. Max depth being none means the node extends until all leaves are pure or all leaf nodes contain fewer samples than min samples split, which is set as two in this work. Min sample leaf is the minimum sample number on leaf nodes. Max leaf nodes are the optimal nodes defined by a relative reduction in purity in the best-first fashion. Max leaf nodes being none means there is no restriction on the number of leaf nodes. As for RNN, the activation function chosen was the most popular non-linear function rectified linear unit (ReLU), expressed as $f\left( x \right) = \max \left( {z, 0} \right)$. As the number of the hidden units becomes larger, the prediction accuracy of RNN slightly increases but the running speed is slowed down. In this case, we choose the number of the hidden units to be 300. Learning rate is typically log-spaced and change of it commonly does not make significant improvement. We choose learning rate to be 10^–3^. Lay number is set to be 2, because two-layer enables RNN more accurate than single-layer in predicting PM_10_, as we’ve tested.

## Results and discussion

### Feature importance of PM_10_

**Figure 1:**
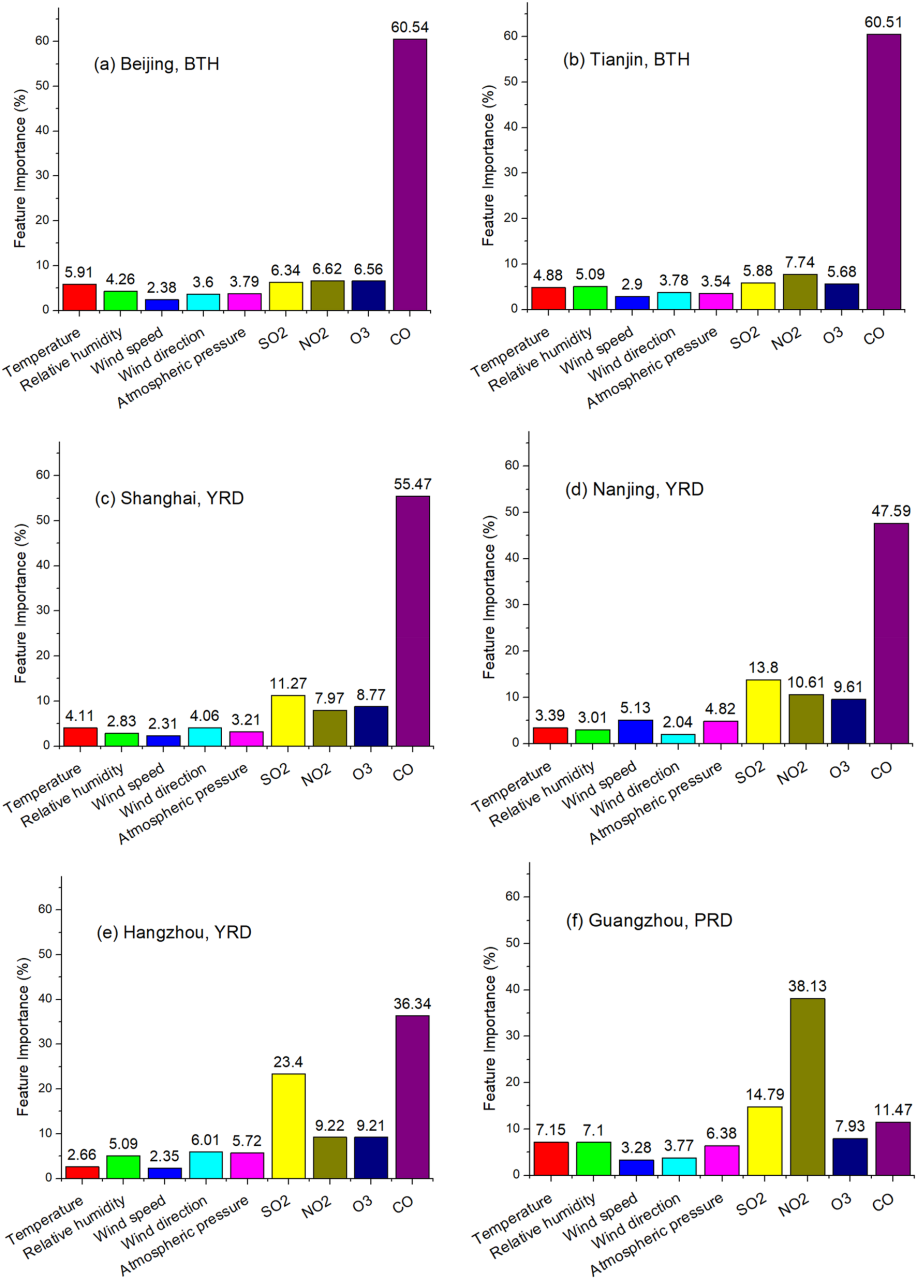

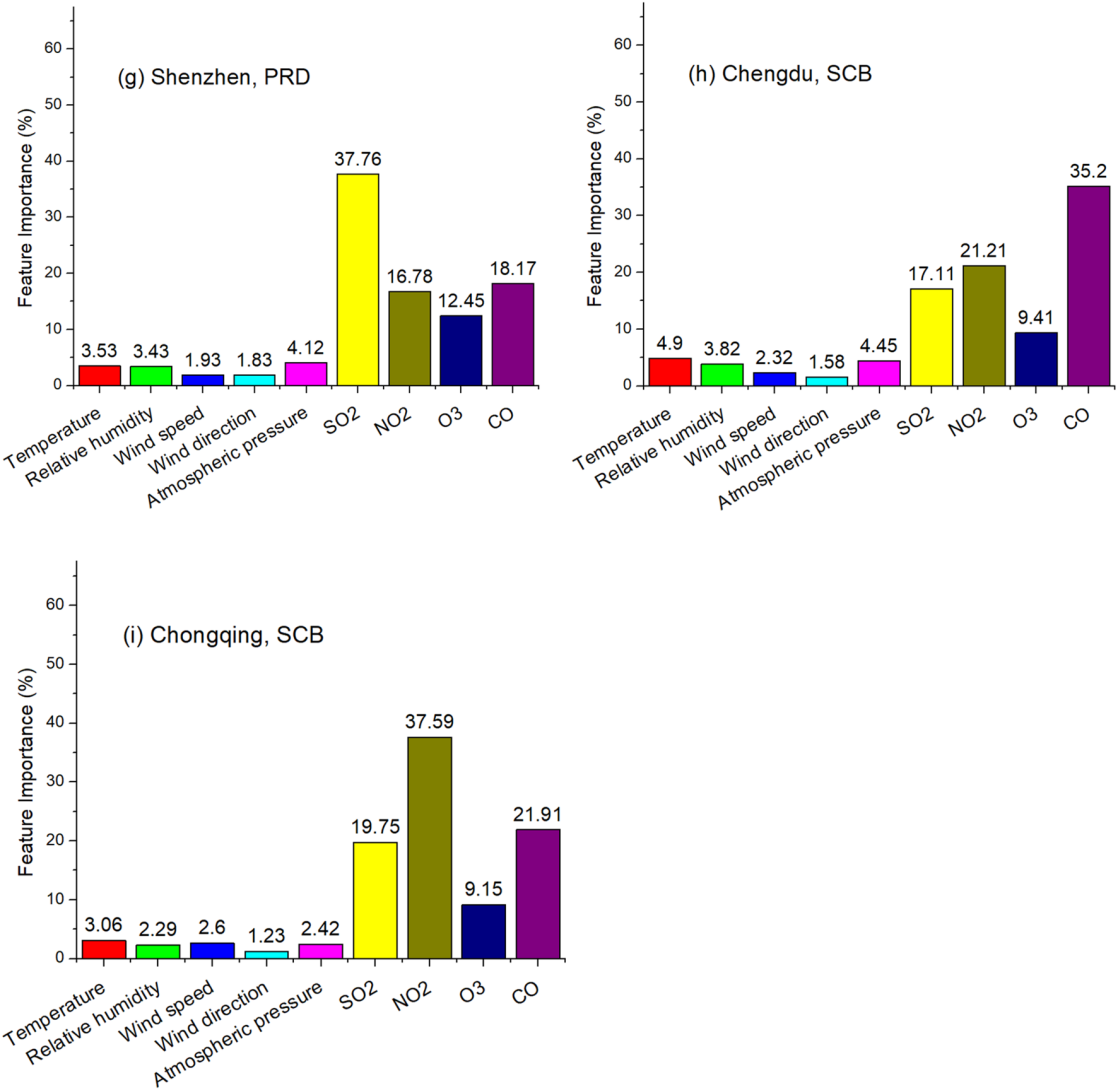
Feature Importance for PM_10_ in wintertime from December 2014 to February 2019.

Feature Importance (FI), calculated by Random Forest, is able to quantify the significance of each input to impact the output. The higher the score that an input gets, the more significant that input is to the output. The hourly meteorological conditions and air pollutants in the wintertime of past more than five years (December 2014 to February 2019) were input to calculate the long-term FI of PM_10_, shown in Fig. 1. First and foremost, Fig. 1 quantitatively demonstrates that gaseous air pollutants (SO_2_, NO_2_, O_3_ and CO) were more significant than the meteorological conditions in shaping PM_10_, as the FI of gaseous air pollutants outscored that of meteorological conditions combined. SO_2_ and NO_2_ were positively correlated with PM_10_, because they were the precursors of sulfate and nitrate, the main components of PM_10_^27^. Tropospheric O_3_ in the surface air and PM_10_ were negatively associated, because PM_10_ is a promoter that speeds up the aerosol sink of hydroperoxy radicals^28^. The strongly positive association between CO and PM_10_ was because they were emitted from same sources, such as coal-base domestic heating and traffic. The possible chemical bonds between CO and PM_10_ need further investigation. As for Beijing and Tianjin of BTH, the influence of CO on PM_10_ was far greater than that of other gaseous air pollutants and NO_2_ contributed more pivotally than SO_2_ for PM_10_. As for Shanghai, Nanjing and Hangzhou of YRD, SO_2_ played a more crucial role than NO_2_ in reproducing PM_10_. The influence of CO on PM_10_ was also predominant in YRD but less critical than that in BTH. As for Guangzhou and Shenzhen of PRD, NO_2_ and SO_2_ had higher FI than CO, revealing a different pattern of PM_10_ in stark comparison with BTH and YRD. As for PM_10_ in SCB, CO and NO_2_ were the primary FI in Chengdu and Chongqing, respectively. Therefore, the spatial heterogeneity of regional PM_10_ in China is corroborated. We then calculate the annual FI for PM_10_ from the aforementioned nine cites, shown in Table 1. Despite of the ebb and flow of FI in some year, the results are consistent for wintertime PM_10_ in a city. CO is associated with the insufficient combustion in the coal-based house heating while NO_2_ is mainly emitted by automotive vehicles, curbing coal-based house heating in BTH/YRD and controlling vehicles in PRD and SCB are the best ways to lower PM_10_.

**Table 1:** FI of wintertime PM_10_ in nine regional core cities in Scenario one.

	SO_2_ (%)	NO_2_ (%)	O_3_ (%)	CO (%)
**Beijing, BTH**				
2014–2015	8.7	6.9	6.3	78.1
2015–2016	13.2	12.8	4.6	69.4
2016–2017	5.2	4.9	4.2	85.8
2017–2018	10.0	9.8	12.6	67.7
2018–2019	6.0	12.7	14.2	67.1
**Tianjin, BTH**				
2014–2015	5.7	13.4	6.3	74.6
2015–2016	9.0	9.8	4.8	76.4
2016–2017	5.8	8.1	5.2	81.0
2017–2018	12.1	11.3	10.3	66.3
2018–2019	7.0	11.4	11.8	69.8
**Shanghai, YRD**				
2014–2015	13.4	5.0	5.8	75.8
2015–2016	50.0	7.6	12.0	30.4
2016–2017	13.4	9.1	11.8	65.7
2017–2018	36.5	5.9	8.1	49.5
2018–2019	25.3	12.3	12.7	49.5
**Nanjing, YRD**				
2014–2015	27.2	6.4	6.6	59.8
2015–2016	18.4	13.7	13.4	54.5
2016–2017	24.1	10.4	9.5	56.0
2017–2018	13.9	8.6	8.8	68.7
2018–2019	18.6	10.8	9.3	61.3
**Hangzhou, YRD**				
2014–2015	44.8	8.4	12.6	34.3
2015–2016	27.3	12.6	9.4	50.7
2016–2017	19.3	32.9	12.3	35.5
2017–2018	20.5	7.7	9.0	62.8
2018–2019	44.5	13.1	15.7	26.7
**Guangzhou, PRD**				
2014–2015	45.6	21.5	7.7	25.2
2015–2016	30.1	45.9	10.3	13.7
2016–2017	51.5	27.2	8.2	13.1
2017–2018	59.3	10.2	8.5	22.0
2018–2019	7.1	64.0	14.6	14.3
**Shenzhen, PRD**				
2014–2015	39.6	28.9	17.3	14.2
2015–2016	35.9	22.5	17.1	24.5
2016–2017	40.9	17.6	12.9	28.6
2017–2018	49.0	18.1	14.2	18.7
2018–2019	37.6	27.8	15.1	19.5
**Chengdu, SCB**				
2014–2015	14.7	50.70	8.1	26.5
2015–2016	49.6	10.6	7.6	32.2
2016–2017	20.3	10.6	10.1	59.0
2017–2018	8.6	26.4	14.0	51.0
2018–2019	28.8	41.4	14.6	15.2
**Chongqing, SCB**				
2014–2015	53.5	18.2	6.5	21.8
2015–2016	20.0	43.5	13.6	22.9
2016–2017	49.3	10.9	7.6	32.2
2017–2018	47.1	24.6	10.5	17.8
2018–2019	20.0	52.3	9.3	18.4

### Prediction of PM_10_ using SO_2_, NO_2_ and CO as inputs

**Figure 2:**
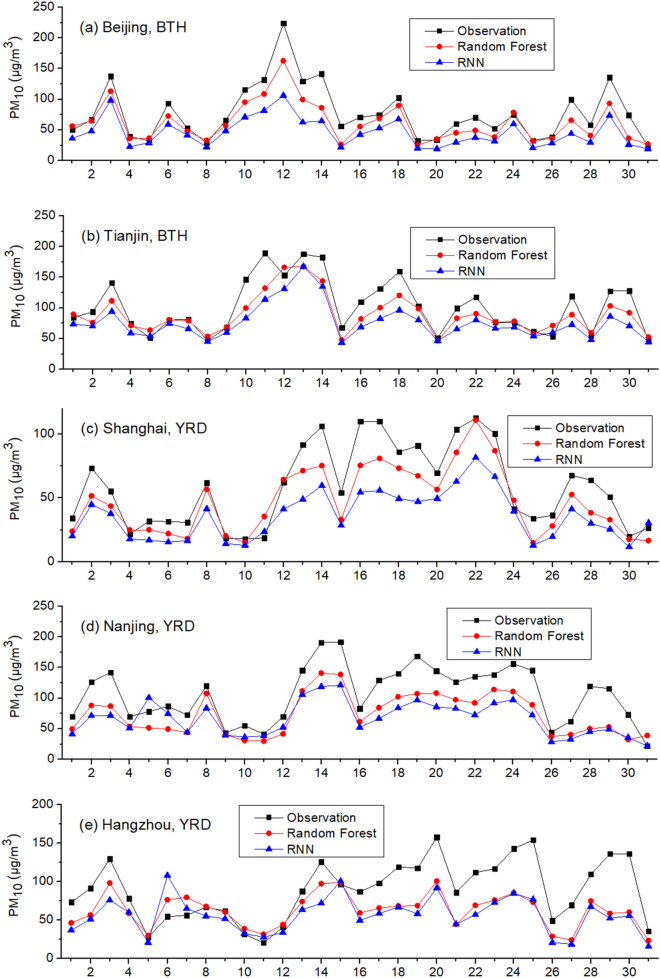

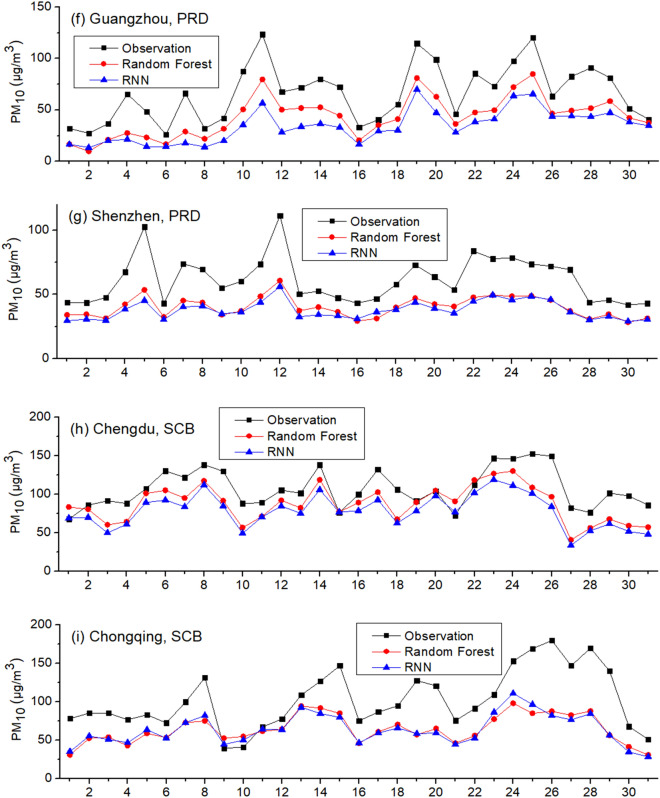
Observed and simulated PM_10_ in January 2019: Scenario one.

Due to the leading roles that gaseous air pollutants (SO_2_, NO_2_ and CO) play in shaping PM_10_, they are used to predict hourly PM_10_ without meteorological circumstances. Training period is set to be December and February while testing period is January (Scenario one). Training and testing data are from the same city. Pearson correlation coefficient (R) and Root Mean Square Error (RMSE) are used as two statistic indicators to evaluate the performance of RF and RNN, and the results are shown in Table 2 and Fig. 2. As Table 2 indicates, both RF and RNN show good accuracy in simulating hourly PM_10_ with only three gaseous air pollutants as inputs. In most cases, the Pearson correlation coefficient (R) between hourly observed and RF/RNN-simulated data is larger than 0.8. RNN is related with time series, as it recursively associates the dataset in the direction of sequence evolution. However, in this case, RNN’s not outperforming Random Forest in all nine cities signals that PM_10_ was not strongly linked to the time series with one hour interval. This finding reveals that, compared with the impact of gaseous pollutants, the concentration of PM_10_ at a given time-point is more relevant to the gaseous air pollutants at the same time than to their previous levels one hour prior. Also, when using the gaseous air pollutants in timestamp (T-1) as inputs, the performances of RF and RNN are slightly worse for predicting PM_10_ in timestamp T, compared with that using the gaseous air pollutants in timestamp (T) as inputs. Moreover, the Pearson correlation coefficient of PM_10_ in timestamp T and concomitant gaseous pollutants in timestamp T is greater than that of PM_10_ in timestamp T and gaseous pollutants one hour prior in timestamp (T-1). This finding not only unravels that PM_10_ and gaseous air pollutants were in thermodynamic dynamic equilibrium, but also implies the formation and deposition of PM_10_ tended to occur in less than one hour. Furthermore, when training data and testing data are extracted from different cities, the prediction accuracy is reduced, implying every city had its own unique pattern of PM_10_.

**Table 2:** Performance of machine learning in predicting hourly PM_10_.

Time	Random Forest		RNN	
	R	RMSE	R	RMSE
**Beijing, BTH**				
2015.1	0.85	56.3	0.86	58.6
2016.1	0.91	60.2	0.92	41.0
2017.1	0.86	77.4	0.88	82.9
2018.1	0.74	34.5	0.75	34.8
2019.1	0.85	35.9	0.85	35.8
**Tianjin, BTH**				
2015.1	0.87	57.9	0.92	51.2
2016.1	0.89	50.5	0.89	50.3
2017.1	0.88	51.9	0.88	51.9
2018.1	0.78	26.4	0.82	23.5
2019.1	0.79	36.8	0.81	42.9
**Shanghai, YRD**				
2015.1	0.86	42.7	0.91	44.2
2016.1	0.83	30.3	0.88	27.4
2017.1	0.79	25.6	0.79	22.6
2018.1	0.86	27.8	0.88	28.7
2019.1	0.85	34.0	0.87	31.9
**Nanjing, YRD**				
2015.1	0.81	59.9	0.84	69.9
2016.1	0.69	48.4	0.84	70.2
2017.1	0.81	32.6	0.97	29.1
2018.1	0.80	53.2	0.81	55.6
2019.1	0.83	44.5	0.83	50.1
**Hangzhou, YRD**				
2015.1	0.89	39.2	0.90	39.7
2016.1	0.84	34.8	0.87	30.9
2017.1	0.80	34.2	0.84	32.4
2018.1	0.82	30.0	0.87	28.3
2019.1	0.61	45.9	0.61	49.7
**Guangzhou, PRD**				
2015.1	0.86	23.5	0.930	16.2
2016.1	0.86	17.5	0.882	16.4
2017.1	0.88	27.9	0.895	29.9
2018.1	0.90	33.5	0.921	32.9
2019.1	0.89	27.9	0.87	38.4
**Shenzhen, PRD**				
2015.1	0.80	23.7	0.79	28.3
2016.1	0.73	16.4	0.75	16.0
2017.1	0.79	12.2	0.80	13.8
2018.1	0.89	17.7	0.90	18.3
2019.1	0.81	25.3	0.81	27.7
**Chengdu, SCB**				
2015.1	0.84	78.1	0.86	77.4
2016.1	0.84	31.5	0.79	36.7
2017.1	0.70	96.7	0.69	107.7
2018.1	0.78	37.6	0.86	31.3
2019.1	0.57	34.9	0.68	36.8
**Chongqing, SCB**				
2015.1	0.82	69.7	0.81	75.1
2016.1	0.60	37.2	0.63	34.8
2017.1	0.79	41.2	0.85	38.6
2018.1	0.80	35.3	0.89	26.6
2019.1	0.64	49.2	0.69	48.6

### Thermodynamic equilibrium between gaseous air pollutants and PM_10_

**Figure 3:**
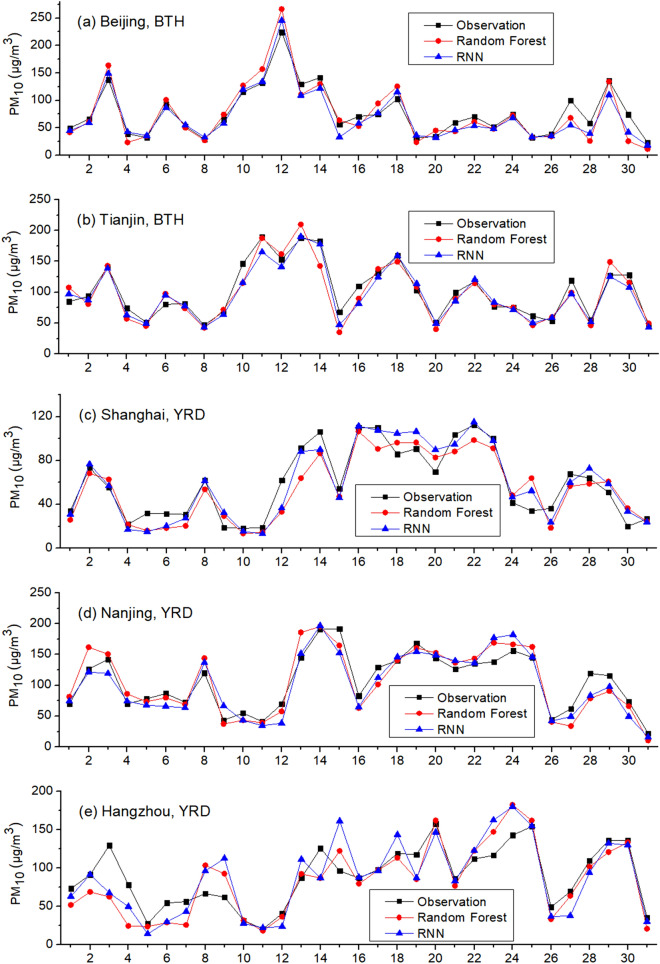

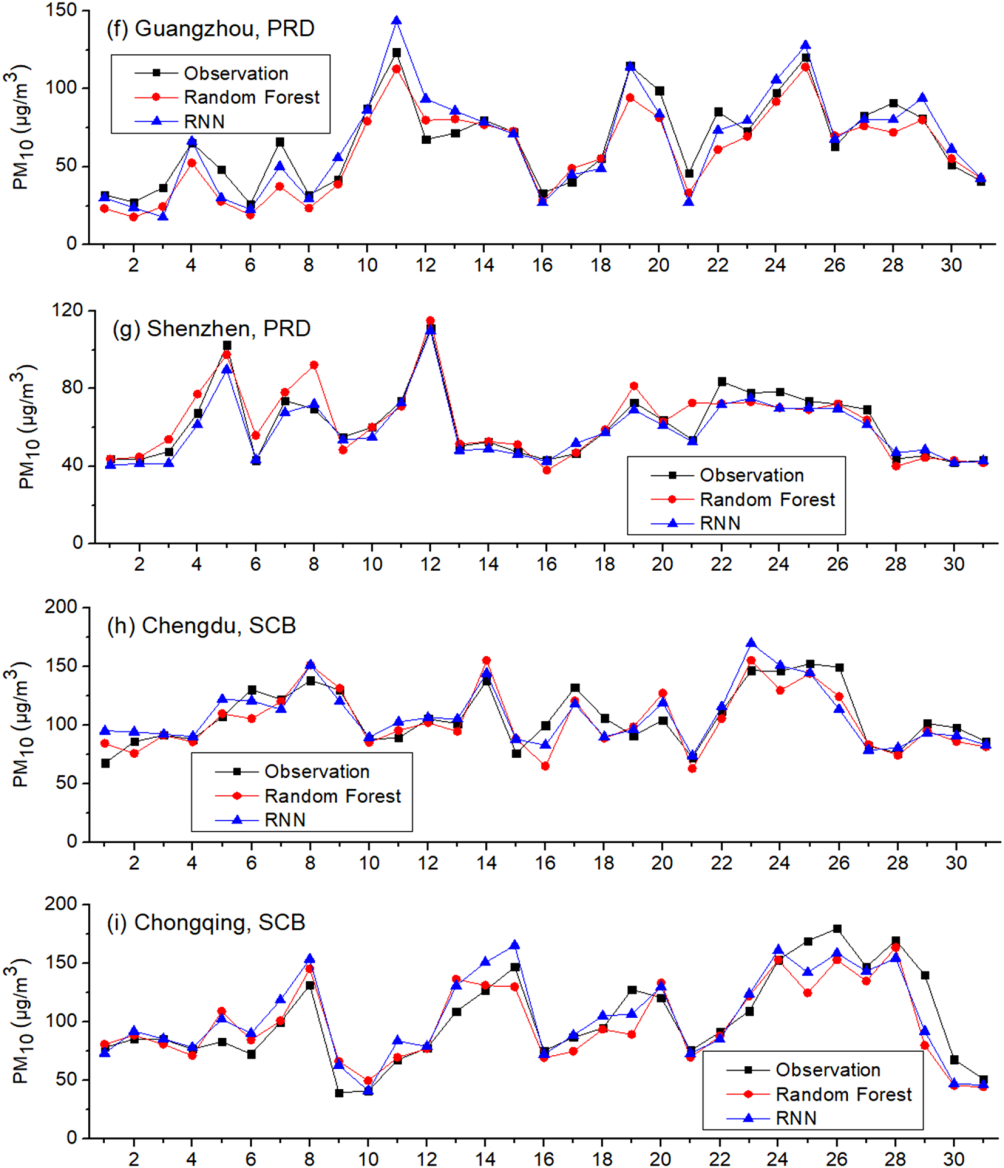
Observed and simulated PM_10_ in January 2019: Scenario two.

As Fig. 2 and Table 3 show, both RF and RNN ubiquitously underestimate PM_10_ in all nine cities in Scenario one. In contrast with Scenario one, Scenario two is set as the testing period is hourly PM_10_ in one day in January 2019 and the training period is hourly PM_10_ in the remaining days in January 2019. Training and testing data are from the same city. Inputs include SO_2_, NO_2_ and CO as well. The results are given in Fig. 3. As Fig. 3 shows, the underestimations do not take place in Scenario two. In addition, we use the gaseous pollutants in January 2018 and December 2017/February 2018 as inputs to train RF and RNN, respectively. The results are similar: the prediction results of PM_10_ in January 2019 using the data in January 2018 for training are greater than that using the data in February 2018 for training. The simulations of RF and RNN both underestimate the PM_10_ level in all nine cities when using the data in December 2017 and February 2018 for training, similar to Scenario one, indicating this is a ubiquitous phenomenon.

**Table 3:** Monthly average observed and predicted PM_10_ in January of 2019: Scenario one.

	Observation (μg/m^3^)	RF-simulated (μg/m^3^)	RNN-simulated (μg/m^3^)
Beijing, BTH	77.2	61.6	45.7
Tianjin, BTH	102.0	88.8	76.2
Shanghai, YRD	59.1	47.4	36.1
Nanjing, YRD	116.6	73.6	66.6
Hangzhou, YRD	89.2	62.5	56.3
Guangzhou, PRD	66.1	43.3	34.0
Shenzhen, PRD	61.6	40.1	37.9
Chengdu, SCB	107.0	87.2	78.4
Chongqing, SCB	102.6	64.3	64.0
Average	86.8	63.2	55.0

Two insidious causes account for this. The major reason is the chemical processes of sulfur dioxide forming sulfate and nitrogen dioxide forming nitrate are exothermic. Since the temperature in January is lower than that in December and February, the thermodynamic equilibrium shifts lopsidedly in favor of augmenting PM_10_ in January. Moreover, indigenous flora plays an important role for the removal of PM_10_^29–32^. As the leaf area index dwindles and the metabolism of trees slows down with the decrease of temperature, the change of phenology of indigenous plants is the minor reason for severity of PM_10_ in wintertime.

### Insignificance of long-range transport

The motivation of this work is partially stimulated by the sizzling debates in several previous studies^33–37^. Guo et al.^33^ inferred that primary emissions and regional transport of PM in Beijing were insignificant in spawning haze. Li et al.^34^ demurred to Guo et al.^33^, insisting that long-range transport was the major cause of severe haze in Beijing. Zhang et al.^35^ contended that the back trajectory analysis by Li et al.^34^ was unsuitable for urban-scale investigations and polluted periods in Beijing were typically linked to stagnant conditions with weak and variable winds. Cao and Zhang^36^ criticized Guo et al.^33^ for ignorance of non-fossil emission sources, such as biomass burning, cooking, and biogenic emissions. Zhang et al.^37^ was opposed to Cao and Zhang^36^, stating that there was little evidence showed that the biogenic source is an ascendant contributor to severe urban PM pollution worldwide. According to Ni et al.^38^, when the horizontal transportation of air pollutants exceeds 300 km, it is considered as long-distance transport.

Machine learning can give an assessment to this argument. The gestations of the haze can be ascribed to crescendo of gaseous precursors, increase of primary emission, or long-range transport. The lifespans of SO_2_ and NOx are short^33^. The gaseous air pollutants and solid PM_10_ have different physical characteristics, making them unlikely to transport together for a long distance. Hence, our theory to judge the causes of the ups and downs of PM_10_ level is: when using gaseous air pollutants (SO_2_, NO_2_ and CO) as inputs, if RF and RNN catch the maximum, the high episodes were induced by the increase of secondary inorganic aerosols or change of primary sources; otherwise, it’s elicited by long-range transport.

The average of the monthly average discrepancy between simulation and observation in Scenario two is less than 15% of the observation. Hence, RF and RNN catch the undulations of PM_10_ using only gaseous air pollutants as inputs, indicating the insignificance of long-range transport. In urban areas of China, fugitive dust from roads, construction sites, and unpaved soil sources normally account for 30%-50% of PM_10_, which is referred as primary PM_10_^39^. CO is a presumable indicator for primary PM_10_. The sporadic sandstorm may induce the long-range transport of PM_10_ from the far-flung deserts in the northwestern China^40^. RF and RNN catch all the fluctuations of PM_10_ using gaseous air pollutants as inputs, indicating the long-range transport induced by spasmodic sandstorm did not occur in January 2019. Thus, we second and shore up the viewpoints of Guo et al.^33^.

## Conclusion

Air pollution has become a hot button in China in recent years. In this work, we take a deeper insight into PM_10_. To wrap up, we deduce the following conclusions. We find that PM_10_ was more statistically correlated to the gaseous air pollutants (SO_2_, NO_2_ and CO) than meteorological conditions. The spatial heterogeneity and temporal homogeneity of PM_10_ in China are quantitatively chronicled, signifying each city had its own unique PM_10_ pattern. RNN and RF are able to accurately predict hourly PM_10_ using only SO_2_, NO_2_ and CO as inputs. The long-range transported PM_10_ was insignificant for haze. The severity of PM_10_ was impacted by the lopsided shift of thermodynamic equilibrium and the phenology of local flora.
